# Dominant-negative signal transducer and activator of transcription (STAT)3 variants in adult patients: A single center experience

**DOI:** 10.3389/fimmu.2022.1044933

**Published:** 2022-12-20

**Authors:** Oded Shamriz, Limor Rubin, Amos J. Simon, Atar Lev, Ortal Barel, Raz Somech, Maya Korem, Sigal Matza Porges, Tal Freund, David Hagin, Ben Zion Garty, Amit Nahum, Vered Molho Pessach, Yuval Tal

**Affiliations:** ^1^ Allergy and Clinical Immunology Unit, Department of Medicine, Hadassah Medical Organization, Faculty of Medicine, Hebrew University of Jerusalem, Jerusalem, Israel; ^2^ The Lautenberg Center for Immunology and Cancer Research, Institute of Medical Research Israel-Canada, Faculty of Medicine, Hebrew University of Jerusalem, Jerusalem, Israel; ^3^ Sheba Cancer Research Center and Institute of Hematology, Sheba Medical Center, Ramat Gan, Israel; ^4^ Pediatric Department A and the Immunology Service, Jeffrey Modell Foundation Center, Edmond and Lily Safra Children’s Hospital, Tel-Hashomer Medical Center, Affiliated to the Sackler Faculty of Medicine, Tel Aviv University, Tel Aviv, Israel; ^5^ The Genomic Unit, Sheba Cancer Research Center, Sheba Medical Center, Ramat Gan, Israel; ^6^ Sheba Medical Center, Wohl Institute of Translational Medicine, Ramat Gan, Israel; ^7^ Department of Clinical Microbiology and Infectious Diseases, Hadassah Medical Center, Faculty of Medicine, Hebrew University of Jerusalem, Jerusalem, Israel; ^8^ Department of Human Genetics, Institute for Medical Research, the Hebrew University of Jerusalem, Jerusalem, Israel; ^9^ Department of Biotechnology, Hadassah Academic College, Jerusalem, Israel; ^10^ Allergy and Clinical Immunology Unit, Department of Medicine, Tel-Aviv Sourasky Medical Center and Sackler Faculty of Medicine, Tel Aviv University, Tel Aviv, Israel; ^11^ Sackler School of Medicine, Tel Aviv University, Tel-Aviv, Israel; ^12^ Felsenstein Medical Research Center, Rabin Medical Center, Petach-Tikva, Israel; ^13^ Allergy and Clinical Immunology Unit, Schneider Children’s Medical Center, Petach-Tikva, Israel; ^14^ Pediatrics Department A, Soroka University Medical Center and Faculty of Health Sciences, Ben-Gurion University of the Negev, Beer Sheva, Israel; ^15^ Pediatric Dermatology Service, Department of Dermatology, Hadassah Medical Organization, Faculty of Medicine, Hebrew University of Jerusalem, Jerusalem, Israel

**Keywords:** stat3, hyper IgE syndrome, adults, inborn errors of imunity, dupilumab

## Abstract

**Background:**

Autosomal dominant hyper-IgE syndrome (AD-HIES) caused by dominant negative (DN) variants in the signal transducer and activator of transcription 3 gene (*STAT3*) is characterized by recurrent Staphylococcal abscesses, severe eczema, chronic mucocutaneous candidiasis (CMC), and non-immunological facial and skeletal features.

**Objectives:**

To describe our experience with the diagnosis and treatment of adult patients with AD-HIES induced by DN-*STAT3* variants.

**Methods:**

The medical records of adult patients (>18 years) treated at the Allergy and Clinical Immunology Clinic of Hadassah Medical Center, Jerusalem, Israel, were retrospectively analyzed. Immune and genetic workups were used to confirm diagnosis.

**Results:**

Three adult patients (2 males; age 29-41 years) were diagnosed with DN-*STAT3* variants. All patients had non-immunological features, including coarse faces and osteopenia. Serious bacterial infections were noted in all patients, including recurrent abscesses, recurrent pneumonia, and bronchiectasis. CMC and diffuse dermatophytosis were noted in two patients. Two patients had severe atopic dermatitis refractory to topical steroids and phototherapy. Immune workup revealed elevated IgE in three patients and eosinophilia in two patients. Whole exome sequencing revealed DN-*STAT3* variants (c.1166C>T; p.Thr389Ile in two patients and c.1268G>A; p. Arg423Gln in one patient). Variants were located in DNA-binding domain (DBD) and did not hamper STAT3 phosphorylation Treatment included antimicrobial prophylaxis with trimethoprim/sulfamethoxazole (n=2) and amoxycillin-clavulanic acid (n=1), and anti-fungal treatment with fluconazole (n=2) and voriconazole (n=1). Two patients who had severe atopic dermatitis, were treated with dupilumab with complete resolution of their rash. No adverse responses were noted in the dupilumab-treated patients.

**Discussion:**

Dupilumab can be used safely as a biotherapy for atopic dermatitis in these patients as it can effectively alleviate eczema-related symptoms. Immunologists and dermatologists treating AD-HIES adult patients should be aware of demodicosis as a possible manifestation. DN-*STAT3* variants in DBD do not hamper STAT3 phosphorylation.

## 1 Introduction

Signal transducer and activator of transcription (STAT)3 is a key transcription factor involved in the function and development of T helper (Th)17 cells (1). Following stimulation with interleukin (IL)-6 and transforming growth factor (TGF)-β, STAT3 is phosphorylated and, together with RORγT, induces differentiation of CD4^+^ T cells into functional Th17 cells. These cells secrete IL-17 and IL-22 and play a critical role in immune responses against fungal and bacterial infections (1).

Dominant negative (DN) heterozygous variants of *STAT3* induce autosomal dominant hyper immunoglobulin (Ig)E syndrome (AD-HIES) (2). This inborn error of immunity (IEI) is characterized by impaired counts and effector functions of Th17 cells and increased susceptibility to fungal and Staphylococcal infections (3). Immune features also include decreased plasma and memory B-cell counts, *via* impaired IL-21-mediated signaling (4) and a shift towards a Th2-mediated immune response, which manifests as atopic dermatitis and eosinophilia (5).

AD-HIES is also characterized by non-immunological features. Coarse faces are a known feature of AD-HIES (6). Furthermore, retained primary teeth can be seen in AD-HIEs patients, as evident in 72% of a previously reported 30-patient cohort (6). IL-4 and other cytokines-mediated bone resorption may induce osteopenia, osteoporosis and frequent bone fractures (6). Other skeletal characteristics of AD-HIES consist of joint hyperextensibility and craniosynostosis. Scoliosis is also seen in up to a third of the patients (6). Finally, rates of vascular anomalies, including brain and cardiac arterial ectasias and aneurysms, were reported in a cohort of 21 adult patients to be as high as 84% and 50%, respectively (7).

In recent years, advances in mechanism-targeted biological treatments have revolutionized the care of patients with IEI and other rare immune-mediated diseases. Different biologic treatments were used in patients with AD-HIES and demonstrated efficacy in relieving symptoms. For example, benralizumab, an anti-IL-5 receptor α chain, monoclonal antibody was used to treat AD-HIES with eosinophilic asthma (8). Omalizumab, an anti-IgE antibody, was also used to treat patients with AD-HIES and was shown to alleviate respiratory symptoms and pulmonary function tests (9). However, there is an increasing amount of reports of AD-HIES patients that were successfully treated with dupilumab, an anti-IL-4/IL-13 receptor α subunit monoclonal antibody (10–17).

Here, we present additional information regarding the clinical course, diagnosis, and treatment of adult patients with AD-HIES, as well as details on the successful treatment of these patients with dupilumab.

## 2 Methods

### 2.1 Study design and patients

This is a retrospective analysis of the medical records of adult patients (age >18 years) with AD-HIES who were treated at the Allergy and Clinical Immunology Unit of Hadassah Medical Center, Jerusalem, Israel. All patients underwent genetic diagnostic tests that confirmed DN-*STAT3* variants. Variant pathogenicity was confirmed by *in vitro* assays. The clinical severity of AD-HIES was evaluated using the National Institute of Health (NIH) score (10). The severity of atopic dermatitis in the AD-HIES patients was assessed by the Investigator’s Global Assessment (IGA) scale (18) and Eczema Area and Severity Index (EASI) score (18).

### 2.2 Immune analysis

Lymphocyte subsets of peripheral blood mononuclear cells (PBMCs) were obtained using flow cytometry. T-cell proliferation capacity was evaluated using a thymidine-based DNA incorporation assay as described previously (19). T-cell stimuli consisted of 6 and 25 µg/mL phytohemagglutinin (PHA) and anti-CD3 antibody. In one patient (P2), data regarding the thymidine-based DNA incorporation assay was available from a previous analysis, which consisted of PHA, concanavalin A (CON A), and pokeweed mitogen (PWM) stimuli.

In addition, the humoral immune response was assessed in patients by testing their immunoglobulin levels and specific IgG antibody titers to past vaccines. Confirmation of AD-HIES was obtained by immunoblot analysis or flow cytometry assays of phosphorylated (p) STAT3 protein or measurements of Th17 counts using flow cytometry.

### 2.3 STAT3 immunoblotting and phosphorylation assays

PBMCs were isolated from patients and healthy controls using Ficoll-Hypaque and stimulated with IL-6 (20-50 ng/mL) for the indicated times at 37°C. Cells were lysed in RIPA buffer and lysates probed with anti-phospho-STAT3 (Y705) and STAT3 (Cell Signaling Technology, Danvers, MA) using standard Western blotting techniques.

For the flow cytometry-based STAT3 phosphorylation assay, PBMCs were stained with anti-CD4 (Pacific Blue clone RPA-T4; BioLegend Cat #300521) before being stimulated with either IL-10 (10n g/mL; Miltenyi Biotec) or IL-21 (100 ng/mL; Miltenyi Biotec) for 20 min at 37°C. Cells were then fixed with 4% paraformaldehyde for 10 min at 37°C and incubated in permeabilization buffer (True-Phos Perm Buffer, BioLegend Cat #425401) overnight at -20°C. The next day, cells were washed and stained with anti-phospho-STAT3 (pY705) (PE clone 13A3-1; BioLegend Cat #651004) for 4 hours at 4°C.

### 2.4 Intracellular IL-17 staining

Frozen PBMCs from patients and healthy controls were thawed and stimulated with phorbol myristate acetate (20 ng/mL) and ionomycin (1 μg/mL) overnight at 37°C in a 5% CO_2_ atmosphere in the presence of Brefeldin A (1 μL/mL of 1000X; BioLegend Cat #420601). After 16 hours, cells were fixed, permeabilized, and stained for CD4 (Pacific Blue clone RPA-T4; BioLegend Cat #300521), IFNγ (AF647 clone 4S.B3; BioLegend Cat #502516), and IL-17 (FITC clone BL168; BioLegend Cat #512304). Cells were acquired using a BD FACSCanto II flow cytometer and data analysis performed using FlowJo software (V10.0, TreeStar). The aim was to evaluate intracellular cytokine staining within CD4^+^ lymphocytes.

### 2.5 Genetic workup

Exome sequencing was performed using the Twist Human Core Exome Plus Kit (Twist Bioscience, San Francisco, CA, USA) on a NovaSeq 6000 sequencing machine (Illumina, San Diego, CA, USA). Paired end reads (2 × 100 bp) were obtained and processed for each sample. The Illumina Dragen Bio-IT Platform version 3.9 was used to align reads to the human reference genome (hg38) based on the Smith-Waterman algorithm (20) and to call variants based on GATK variant caller version 3.7 (21). Additional variants were called with Freebayes version 1.2.0 (22). Variant annotation was performed using KGG-Seq version 1.2 (23). Further annotation and filtration steps were performed by in-house scripts using various additional datasets.

### 2.6 Ethical review of the study

This study was approved by the institutional review board (IRB) of Hadassah Medical Center (IRB number: HMO-0473-22). A waiver for participant consent was gained by the IRB of Hadassah Medical Center.

## 3 Results

### 3.1 Patient characteristics

Clinical characteristics of the patients are presented in **Table 1**. Three adult patients with AD-HIES due to DN-*STAT3* (two males and one female) were followed at our Allergy and Clinical Immunology Unit during the study period. Patient ages at symptom onset ranged from 2 months to 20 years. All patients were genetically diagnosed with DN-*STAT3* in adulthood (21-41 years).

**Table 1:** Clinical characteristics of adult patients with dominant-negative (DN)-*STAT3* variants.

Pt	Age at onset/genetic diagnosis/current age(years)	Gender/ethnicity	Consanguinity/Family history	*STAT3 LOF Heterozygous* variant	Clinical presentation						Antibiotic and antifungal treatment	Biological treatment (dose)	Duration of dupilumab treatment (months)	Outcome/follow-up period(years)
					NIH score	Infectious			Dermatological	Non-immunologic				
						Bacterial	Fungal	Viral						
P1	3 months 24/37	M/J	No/Yes	c.1166C>T, p.Thr389Ile (missense)*	55	Recurrent Abscesses, Bronchiectasis, Recurrent pneumonia with *Haemophilus influenzae* in BAL	Tinea corporis, Onychomycosis, Candida esophagitis	None	Severe AD, Rosacea; demodicosis	Coarse face; Retained primary teeth	Trimethoprim/ sulfamethoxazole, fluconazole itraconazole, terbinafine, voriconazole	Dupilumab/ loading dose 600 mg followed by 300 mg every 2 weeks	17	Resolution of AD/37
P2	2 months 21/29	F/J	No/Yes	c.1166C>T, p.Thr389Ile (missense)*	48	Recurrent pneumonia and OM Recurrent MSSA breast abscesses, skin abscesses Bronchiectasis pneumatocele chronic sinusitis	Oral candidiaisis Tinea corporis and tines pedis	Varicella pneumonia; CMV pneumonia	Seborrheic dermatitis, Severe AD, Rosacea; demodicosis	Coarse face. Osteoporosis and recurrent skeletal fractures, Fat embolism following fracture	Amoxicillin/ Clavulanic acid, fluconazole	Dupilumab/ loading dose 600 mg followed by 300 mg every 2 weeks	7	Resolution of AD/29
P3	20/41/41	M/A	Yes/No	c.1268G>A, p. Arg423Gln, (missense)**	37	Recurrent MSSA. abscesses (liver, prostate, pyomyositis, hidradenitis suppurativa), Recurrent otitis externa, Multifocal pneumonia, Bronchiectasis	None	None	None	Coarse face. Spondylosis, peripheral neuropathy	trimethoprim/ sulfamethoxazole	None	None	Alive/1

Pt, Patient; F, Female; M, Male; A, Arab; J, Jew; AD, Atopic dermatitis; MSSA, Methicillin Sensitive Staphylococcus aureus; BAL, Bronchoalveolar lavage; OM, Otitis media; CMV, Cytomegalovirus; PUVA, psoralen and UVA therapy.

*Patients were previously reported by Woellner et al. (24) and Scheuerman et al. (25). Variants were also previously reported by Minegishi et al. (2) and Crosby et al. (5) **STAT3 Variant was previously reported by Holland et. al (26) and Asano et al. (27).

Patient 1 (P1) and P2 were briefly reported on by Woellner et al. (24) and Scheuerman (25) et al.; they are siblings born to a non-consanguineous Ashkenazi Jewish family harboring three generations of AD-HIES patients. The mother died at 42 years old from pulmonary emboli. She had seven children, and one of them died from pneumonia at the age of 3 months without a definitive diagnosis of AD-HIES. P1 has two children with AD-HIES.

**Figure 1:**
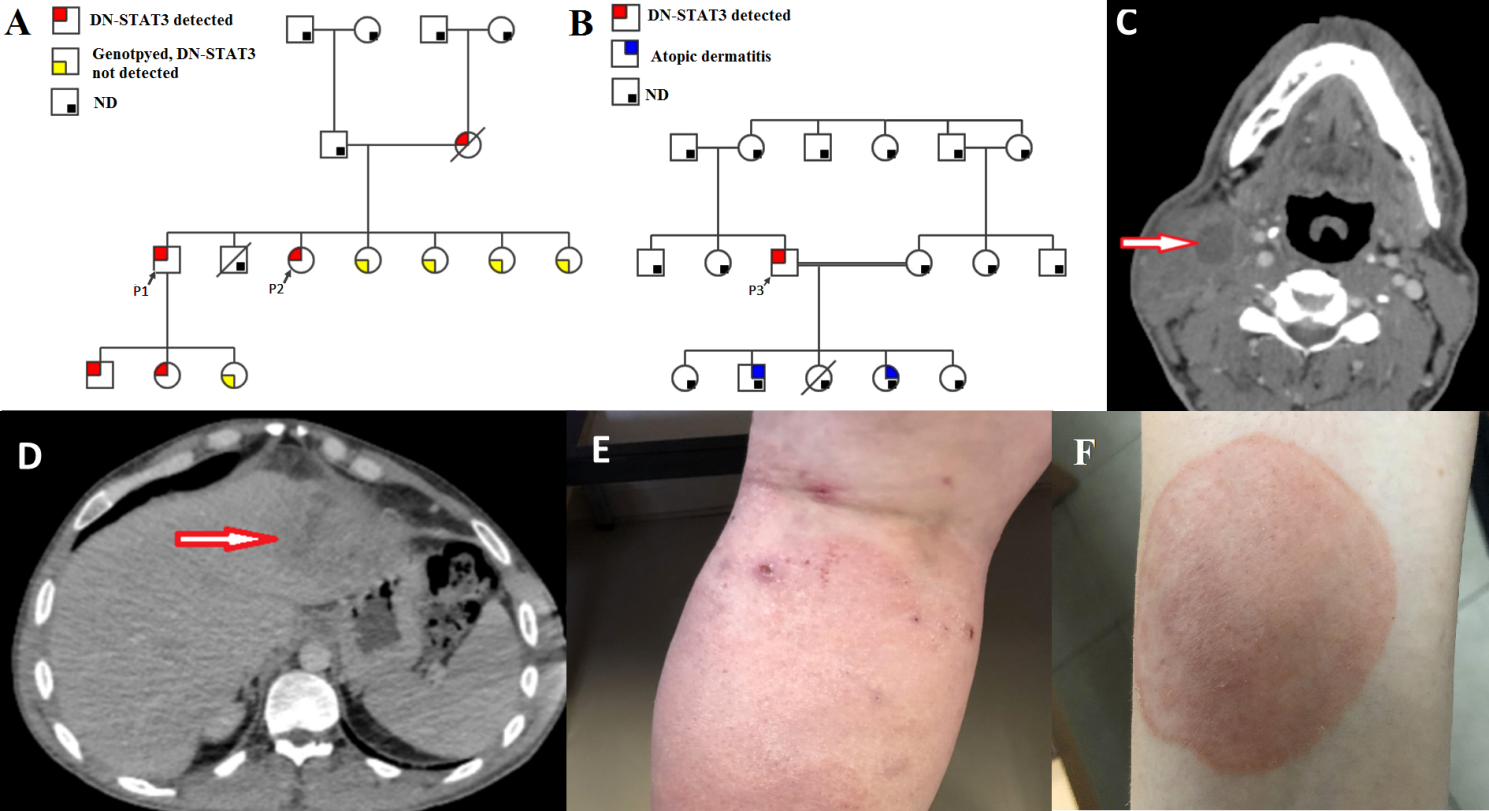
Clinical characteristics of the patients. **(A, B)** Family pedigrees of P1-P2 and P3, respectively, demonstrative of an autosomal dominant inheritance. In red, patients that were genotyped for *STAT3* and were found to harbor DN-*STAT3* variants; In yellow – Subjects that were genotyped and were not found to harbor the DN-*STAT3* variant; In grey – Subjects that were not genotyped and therefore presence of DN-*STAT3* variant cannot be determined or ruled out. ND- not determined. Note patients within 3 generations in the family of P1-P2. DN-STAT3- Dominant negative STAT3 variant. **(C)** Computed tomography (CT) scan of cervical spine of P3, axial view, demonstrating a collection with septations in the sternocleidomastoid muscle measuring 20*41*14 mm with reactive muscle and soft tissue edema. **(D)** Abdominal CT scan of P3, coronal view, demonstrating multiple hypodensities in the left liver lobe (segment 2 and 3) and a hepatic abscess measures 8.2*7.9 cm. **(E)** Atopic dermatitis, as seen in P2. **(F)** Tinea corporis, as seen in P2.

P3 was born to a consanguineous Arab family. He has two children with chronic facial rashes compatible with atopic dermatitis who have not yet been evaluated. The family pedigrees of the patients are presented in **Figure 1**. Although he had course facial features compatible with AD-HIES since childhood, there is no record of significant infections before the age of 20 years. At that age, he presented with severe pneumonia that had a prolonged course and required intravenous antibiotics. At the age of 35 years, he was diagnosed with a liver abscess with growth of *Staphylococcus aureus*, which has resolved with antibiotics. He then presented to our clinic at the age of 41 years, with recurrent pyomoyositis for further immune investigation.

NIH scores for clinical severity of AD-HIES were 55, 48, and 37 in P1, P2, and P3, respectively. All patients had typical infectious manifestations consisting of myositis (**Figure 1C**) and recurrent Staphylococcal abscesses of the skin, breast, and liver (**Figure 1D**). Serious bacterial infections also included recurrent sinopulmonary infections and bronchiectasis in all three patients. Pneumatocele was noted in the history of P2. Dermatological manifestations in P1 and P2 involved severe atopic dermatitis (**Figure 1E**). These patients also had papulopustular rosacea due to demodex, which was observed by dermoscopy. Chronic mucocutaneous candidiasis (CMC) was noted in P1 and P2 and manifested as persistent oral candidiasis, diffuse tinea corporis (**Figure 1F**), and onychomycosis. Severe viral infections occurred in P2, who had cytomegalovirus (CMV) and varicella zoster virus (VZV) pneumonias.

Non-immunological manifestations occurred in all patients. These consisted of coarse facial features in P1-P3, retained primary teeth in P1, and osteoporosis with recurrent skeletal fractures in P2. None of the patients had scoliosis.

### 3.2 Immune workup

Patients were evaluated as adults in our clinic. Their immune workup is presented in **Table 2**. Eosinophilia was noted in P1 and P3 (0.9 and 1 x10^9^/L, respectively; normal: <0.6 x10^9^/L). Lymphocyte subsets revealed inverted CD4^+^/CD8^+^ ratios in all patients. B-cell counts were within normal range in all patients. Lymphocyte proliferation studies were available for P1 and P2 but were normal in both.

**Table 2:** Immune workup of adult patients with DN-*STAT3* variants.

Parameter			P1 (37 yo)	P2 (29 yo)	P3 (41 yo)	Normal Range
Absolute lymphocyte count (10^9^/L)			2.6	2.7	3.5	1.1-3.5
Absolute eosinophil count (10^9^/L)			**0.9**	0.49	**1**	0.0-0.6
Lymphocyte subpopulations (% of total lymphocytes)	T cells	CD3^+^	74	84	84	60-85
		CD4^+^	44	46	48	36-63
		CD8^+^	22	36	33	15-40
		CD4^+^/CD8^+^ Ratio	*0.5*	*1.28*	*1.4*	1.5-3.3
	NK cells	CD56^+^	7	7	19	6-30
	B cells	CD20^+^	15	9	8	5-25
Lymphocyte proliferation study			Normal	Normal	NA	–
Serum immunoglobulins		IgG (mg/dL)	**1562**	**1666**	1185	639-1349
		IgA (mg/dL)	172	151	214	70-312
		IgM (mg/dL)	130	155	57.6	56-352
		IgE (U/mL)	**13500**	**50700**	**25000**	3-100
Specific IgG antibodies		Measles (AU/mL)	**6.20**	**1.1**	**>300**	≥1.1
		VZV (AI)	NA	**2.7**	NA	≥1.1
		Rubella (IU/mL)	**>100**	*19*	**253**	>30
		Mumps (AI)	**2.30**	*0.7*	**19**	>1.1
		HBV surface (mU/mL)	**0.07**	Neg	**62.1**	>0.05
		HAV	**Pos**	Neg	**Pos**	0
		Diphtheria (IU/mL)	NA	*0.03*	NA	≥0.1
		Tetanus (IU/mL)	NA	**0.39**	NA	>0.1
		EBV EBNA (S/CO)	*0.12*	**Pos**	14.82	0-20
		EBV VCA (S/CO)	**28.15**	NA	**16.73**	≥1
		CMV (AU/mL)	**>250**	**>250**	**>250**	>20

NA, Data is not available; Pos, Positive; Neg, Negative; NK, Natural killer; CPM, Counts per minute; PHA, Phytohemagglutinin; PHA6, PHA 6 µg/mL; PHA25, PHA 25 µg/mL; Ig, Immunoglobulins; HBV, Hepatitis B virus; HAV, Hepatitis A virus; CMV, Cytomegalovirus; VZV, Varicella Zoster Virus EBV, Epstein-Barr virus; EBNA, Epstein-Barr nuclear antigen; VCA, Viral capsid antigen. In bold- values above normal range; Italics – below normal range.

All patients had non-diminished IgM, IgA, and IgG levels. IgE was markedly increased in all patients, ranging from 13,500 to 57,000 IU/mL (0-100 IU/mL). Specific IgG production to past vaccines was intact (**Table 2**) and positive IgG titers were noted for CMV and Epstein-Barr virus (EBV) in all patients.

### 3.3 Genetic diagnosis

**Figure 2:**
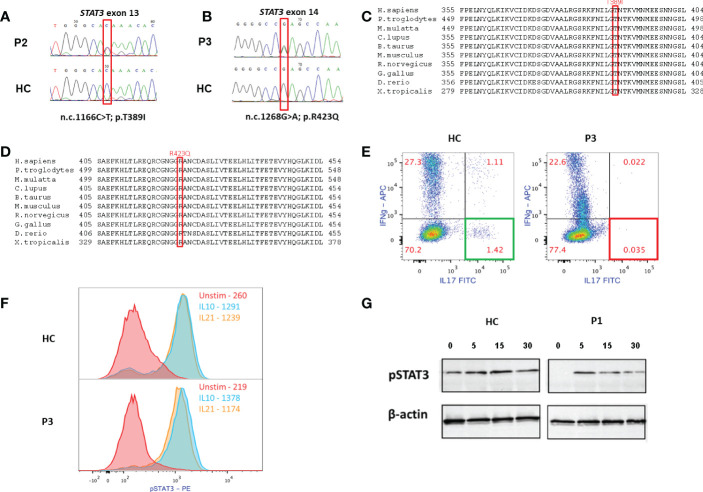
Immune and genetic analyses of the patients. **(A, B)** Sanger sequencings of the *STAT3* variants of P2 and P3, respectively (HC- healthy control). **(C, D)** *STAT3* variants in P1-P2 and P3, respectively, are in evolutionary preserved sites. **(E)** Overnight stimulation of PBMC of P3 with phorbol myristate acetate (PMA) and Ionomycin shows normal production of interleukin (IL)-17 in HC CD4^+^ T cells (left) and near absent IL-17 production in the patient’s CD4^+^ T cells. **(F)** P3’s cells were stimulated with either IL-10 (10ng/mL) or IL-21 (100ng/mL) for 20 minutes. Cells were then fixed, permeabilized and stained for phosphorylated (p) STAT3 Y705. Both HC’s and P3’s CD4^+^ T cells showed normal STAT3 phosphorylation, as can be expected in cases of variant located at the DNA binding domain (DBD). **(G)** Immunoblotting of P1, demonstrating levels of pSTAT3 in T=0,5,15, and 30 minutes following IL-6 stimulation. While there might be decreased expression of pSTAT3 at baseline, P1’s cells did show STAT3 phosphorylation following stimulation, which is compatible with location of the *STAT3* variant in the DBD.

All patients were found to harbor previously reported heterozygous missense variants in the DNA-binding domain (DBD) of *STAT3*; P1 and P2 had c.1166C>T, p.Thr38Ile (2, 5, 24, 25), and P3 had c.1268G>A p.Arg423Gln. The latter variant was previously associated with AD-HIES in 2 families (26), and its pathogenicity was validated in another study (27). The variants found in the three patients were predicted to be pathogenic. The combined annotation dependent depletion (CADD) scores for the variants in P1/P2 and P3 are 29 and 32, respectively, and the frequencies (SeenB4) of the variants are 0 and 1 out of 7934 cases, respectively. Variants’ frequencies in GnomAD of P1/P2 and P3 were 0% and 0.000657% (1 out of 152210 alleles), respectively. Sanger sequencing of these *STAT3* variants is presented in **Figure 2**. The *STAT3* variants are found at evolutionarily preserved sites in all three patients, as seen in genetic analysis from humans to frogs (**Figures 2C, D**, respectively).

P3 had a late and relatively mild presentation of AD-HIES. Unfortunately, hair or nail samples from P3 were not available for Sanger sequencing. Thus, genetic diagnosis of the DN-*STAT3* variant was completed using peripheral blood and cannot rule out that this patient is a mosaic. Moreover, due to a lack of compliance, family segregation studies of P3, including Sanger sequencing of his two children with atopic dermatitis for the DN-*STAT3* variant, are currently not available.

### 3.4 Functional confirmation of variant pathogenicity

The pathogenicity of the identified variants was confirmed by functional studies and included either flow cytometry-based evaluation of CD4^+^ IL-17^+^ T cells (Th17 cells), or Western blot and staining using anti-Y705 phospho-STAT3 antibody. Flow cytometric analysis of PBMCs showed that, compared to a healthy control, Th17 cells were nearly absent in P3 (**Figure 2E**). Though there may have been decreased expression of pSTAT3 at baseline, patients’ cells did show STAT3 phosphorylation (**Figures 2F, G**). This finding is in accordance with the location of the identified variants within the DBD, which would allow normal phosphorylation due to an intact SH2 domain but abnormal STAT3 signaling due to mutations in the DBD. The pathogenicity of the variant (c.1166C>T, p.Thr389Ile) identified in P1 and P2 was demonstrated previously (2, 4, 5, 13). The abnormal IL-17 expression confirms the pathogenicity of the variant (c.1268G>A p.Arg423Gln) identified in P3.

### 3.5 Treatment and outcome

Treatments are presented in **Table 1**. All patients are alive and were treated with prophylactic antibiotics, either trimethoprim/sulfamethoxazole (n=2) or amoxycillin-clavulanate (n=1). P1 and P2 received fluconazole treatment for CMC and severe dermatophyosis. P1 was also treated with itraconazole and terbinafine due to tinea corporis, but without improvement. He also received a course of voriconazole with partial but significant resolution of his tinea corporis.

P1 and P2 had severe atopic dermatitis. P2 had multiple erosive nodules on the face and extremities, consistent with lesions of prurigo nodularis, which are common in patients with severe atopic dermatitis. Both patients failed treatment with potent topical steroids. P1 was also treated with narrow band UVB phototherapy with no improvement of the rash and pruritus. Considering these patients’ impaired immune systems, we were reluctant to treat them with systemic immunosuppressive agents. Thus, subcutaneous injections of dupilumab were initiated at a dose of 300 mg every 2 weeks after a loading dose of 600 mg.

**Figure 3:**
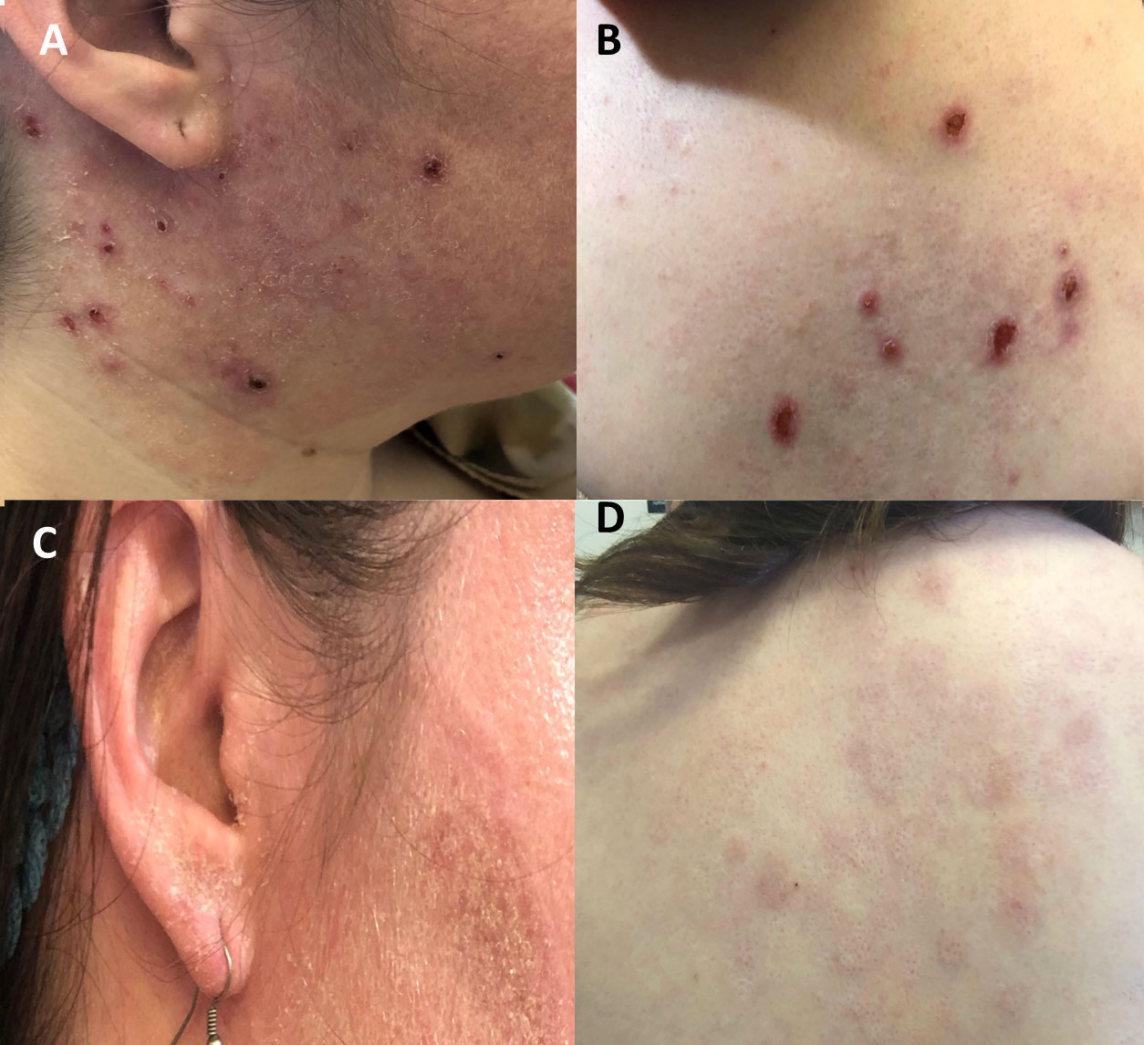
Response to treatment with dupilumab in patients with DN-*STAT3* variants. **(A, B)** Baseline status of atopic dermatitis in P2 with multiple erosive nodules, consistent with prurigo nodularis **(C, D)** Following 3 months of dupilumab treatment, atopic dermatitis eczema has completely resolved.

Duration of dupilumab treatment in P1 and P2 was 17 and 7 months, respectively. Following treatment, complete resolution of the atopic dermatitis rash and pruritus was seen in both patients (**Figures 3A–D**). Atopic dermatitis scores available for the patients consisted of the IGA (P1 and P2) and EASI scores (P2). IGA for P1 before and after 1 year of dupilumab treatment was 3 and 0, respectively. P2 had baseline EASI and IGA scores of 20 and 4, respectively. Following 3 months of dupilumab treatment, both scores markedly decreased, to 1 and 0, respectively In addition, examination of serum IgE levels following dupilumab treatment was available in P1. A significant reduction was observed from baseline (13,500 to 953 U/mL; 3-100 U/mL) following 17 months of treatment. No adverse responses were noted in the dupilumab-treated patients.

## 4 Discussion

In this study, we describe three adult patients with AD-HIES induced by DN-*STAT3* variants. Regarding treatment, dupilumab was shown in our adult-patient cohort to be a novel treatment for atopic dermatitis in AD-HIES, which was both effective and safe.

Our patients were genetically diagnosed in adulthood. P1 and P2 had delayed genetic diagnosis, though clinical onset of symptoms was at infancy. However, P3 presented with disease manifestation at 20 years of age. A recently published review did not mention variants in DN-*STAT3* as possible triggers of monogenic adult-onset IEI, unlike STAT3 gain-of-function (GOF) mutations, which have been reported to induce immune dysregulation and predispose adults to cancer (23). Thus, our report further emphasizes the need for awareness and a high index of suspicion towards IEI by physicians treating adult patients. “Red flags” for IEI, either in the history or findings on physical examination, even if the treated patients are undiagnosed adults, should trigger prompt immune and genetic workups. This will allow early diagnosis, better patient care, and genetic counseling for family members.

Supportive therapy of AD-HIES consists of antibiotics and anti-fungal treatments, either as prophylaxis or upon signs of infections. Other therapies consist of immunoglobulin replacement treatments, by intravenous (IVIG) or subcutaneous (SCIG) routes (28). Use of hematopoietic stem cell transplantations (HSCT) in AD-HIES patients is currently increasing. A recent review of 14 AD-HIES patients demonstrated high rates of long-term survival, as well as resolution of respiratory, dermatologic, and immune features of the disease (29). Osteoporosis and recurrent fractures in AD-HIES, as seen in P2 from our cohort, can be treated with bisphosphonates. It is a result of increased osteoclast quantities and bone resorption due to abnormal IL-6–mediated differentiation (29). Regarding treatment of our patients, none had humoral defects. Apart from the accepted antimicrobial prophylaxis, two patients were successfully treated with dupilumab. One patient with recalcitrant tinea corporis responded well to voriconazole, a second-generation triazole approved for systemic mycoses that has been reported to be effective against dermatophytes *in vitro*, and its use is considered a last resort in unresponsive patients with tinea infections (30).

Previously published cohorts of adult patients with DN-*STAT3* variants are scarce. Some adults with AD-HIES are characterized by normalized IgE levels and degenerative joint diseases (29). Rates of delayed diagnosis are not available in the literature. Interestingly, P3 had a delayed diagnosis of 21 years, since he first presented with severe pneumonia at the age of 20 years, until genetic diagnosis was made. However, delay in diagnosis is expected to decrease in current days, with increasing awareness to disease presentation and wide use of next generation sequencing and molecular diagnosis. Previous life expectancy reports are estimated by a median of 20.5 years and a maximal expectancy of 40 years (29). Major cause of death of adults with AD-HIES is attributed to pulmonary infections and their complications (29). Our patients’ current ages range between 29 and 41 years. All patients in our cohort had recurrent pneumonias and currently they all have bronchiectasis, as seen in CT scans. One patient (P2) also had pneumatocele. Hopefully with improved supportive care and monitoring, survival rates of these patients will increase, as previously suggested by others (29).

The clinical manifestations of our patients are consistent with AD-HIES. Susceptibility to Staphylococcal and fungal infections, high incidence of abscesses, recurrent sinopulmonary infections, bronchiectasis, and CMC are known features of AD-HIES (31). Non-immunological features, such as retained primary teeth, coarse faces, and osteoporosis, were also previously reported (31). Other non-immunologic features, such as scoliosis, are not found in our patients.

All the patients had non-diminished levels of pSTAT3, as demonstrated by immunoblotting or flow cytometry. This is compatible with the location of the variants in the DBD, suggesting decreased function of STAT3 without hampering its phosphorylation. A reduced Th17 subpopulation in P3, despite normal levels of pSTAT3 in flow cytometry, supports this notion and confirms the variant’s pathogenicity. This observation was previously demonstrated in other studies (26).

P3 is notable in our cohort, as he was diagnosed in adulthood and had no reported CMC or recurrent infections during childhood. Indeed, we have not ruled out somatic mosaicism in P3. Intermediate phenotype of 2 patients with DN-*STAT3* variants was previously reported. These patients had non-reduced levels of Th17and a milder clinical presentation, although CMC was noted (32). Suspicion of DN-*STAT3* in P3 was raised due to recurrent abscesses in his liver and striated muscles, as well as coarse facial features. Interestingly, two of his children are currently being evaluated due to atopic dermatitis. However, due to lack of compliance, Sanger sequencings of the children for the relevant DN-*STAT3* variant is currently not available. Thus, the diagnosis of AD-HIES in P3 should be considered even with a lack of classical childhood infections.

A unique feature in two of our patients (P1 and P2) is papulopustular rosacea due to demodicosis. To the best of our knowledge, our study is the first report of chronic demodicosis in DN-*STAT3*. Demodicosis was not originally reported by the international study group of STAT1 GOF (33). Recently, our group described chronic demodicosis in patients from two families with *STAT1* GOF variants (19). Chronic demodicosis has also been reported in other patients with *STAT1* GOF (34–36).

The underlying immune pathogenesis in chronic demodicosis is not well defined. It probably consists of an impaired innate immune response *via* Toll-like receptor (TLR)-2 and decreased counts and effector function of Th17, among other immune pathways (12). In *STAT1* GOF, overexpressed cytokines, such as interferon (IFN)-γ and IL-27, inhibit the function of Th17 *via* suppression of IL-17 and RORγT (37). This mechanistic pathway of impaired Th17-mediated immunity is common to both *STAT1* GOF and *STAT3* loss-of-function and may account for the chronic demodicosis diagnosed in our patients.

STAT3 has a role in regulating Th2 inflammation. This is seen in over-activation of STAT3, which inhibits GATA3, impairing the differentiation of inflammatory T helper cells into Th2 cells and reducing their activities (38). The eosinophilia and atopic dermatitis in our patients are suggestive of increased Th2 function *via* elevated production of IL-5 and IL-4, respectively. Therefore, dupilumab has enabled successful treatment of the patients’ atopic dermatitis by manipulating the underlying immune mechanism without the need for systemic immunosuppressive or cytotoxic drugs. The dose of dupilumab was decided according to current indications for atopic dermatitis in non-HIES patients, with adverse events noted in none of the patients.

It is plausible to suspect that decreasing the Th2-mediated pathway will result in enhanced function of Th1, reducing susceptibility to severe infections. Severe viral infections were noted in P2, suggesting decreased Th1 activity. However, the effect of dupilumab on the Th1-mediated response and secreted cytokines was not evaluated in our study. Nevertheless, Th1 enhancement following dupilumab treatment has been demonstrated in AD-HIES (14).

We further reviewed the literature on reported AD-HIES patients with DN-*STAT3* variants who were treated with dupilumab (**Table 3**). Our search yielded 22 patients (11 males, age 9-42 years) with a mean duration of dupilumab treatment of 11.5 (2–15) months. All of the patients were reported to clinically improve following dupilumab treatment, independent of their *STAT3* variant or age and with no severe adverse reactions. As in our patient, dupilumab doses ranged from 200 to 300 mg per injection every 2 or 4 weeks.

**Table 3:** Previously published dupilumab-treated patients with AD-HIES due to DN-*STAT3* variants.

Number of Reported Patients	Age at dupilumab initiation(years)	Gender	Clinical Manifestations(Baseline SCORAD score)	*STAT3 LOF Heterozygous* variant	Dupilumab Therapeutic regimen (mg)	Outcome (SCORAD following dupilumab)	Duration of dupilumab treatment (months)	Ref.
2	29 and 37	M, F	Recurrent skin and pulmonary infections (NA)	c.1166C>T, p.Thr389Ile	Loading dose 600 mg followed by 200 mg every 2 weeks	Resolution of AD (NA)	7 and 17	Current report
1	2.5	F	Recurrent skin and pulmonary infections (NA)	1144 C>T, p.Arg382Trp	300 mg every 4 weeks	Resolution of AD (NA)	6	(10)
1	17	M	Severe AD, recurrent staph infections, allergy to inhalants (45)	c.1150T>C. p.F384L	300 mg every 2 weeks	Resolution of AD (28/103)	12	(14)
1	9	M	Generalized pruritic erythematous papules and Xerosis; eczema, oral candidiasis, furuncles, pneumatoceles, recurrent pneumonia, abscess in liver and gingiva, knee and finger joint deformities since birth (73)	c.1145G>A, p.R382Q	Loading dose of 200 mg followed by 100 mg every 2 weeks.	Resolution of AD (0)	10.5	(12)
3	9	3-M	PJP, chronic OM(58-78)	c.21323C>G	Loading dose 300 mg every 2 weeks, followed by injections every 28 days	Mild dermatitis in all patients after 4 months (5-15).	4	(13)
			Pneumonia (NA)	p.V343L				
			Neonatal pneumonia, chronic OM (NA)	p.R32Q				
1	21	M	AD, recurrent skin and respiratory tract infections, refractory diarrhea and recurrent colon perforations (NA)	Int10(-2)A>G	300 mg every 3 weeks	Resolution of skin and GI manifestations (NA)	6	(11)
1	33	F	Pruritic eczema, dermatitis (67.45)	1907C > T, p.S636F	loading dose of 600 mg followed by 300 mg every 2 weeks	Resolution of AD (< 10)	10	(17)
1	14	M	Severe AD and EoE (NA)	c. 1294 G>A. p.V432S	Loading dose of 600 mg followed by 300 mg every 2 weeks	Resolution of AD (10)	2	(15)
13	10-42	9-F 4-M	ABPA, AD, Asthma (53.85, 80.3)	c.1144 C > T, p. R382W	200 or 300 mg every two weeks	Resolution of AD (0)	15	(16)
				c.1003 C > T, p.R335W				
				c.2137 G > T, p.V713L				
				c.1979 T > C, p.N660T				
				c.1110(-2) A-G				
				c.1864 A > G, p.T622A				
				c.1388 T > A, p.V463E				
				c.1962_1964delATC,p.I654del				
				c.1144 C > T, p.R382W				
				c.1861 T > G, p.F621V				
				c.1909 G > A, p.V637M				
				c.1003C > T, p.R335W				
				c.1110(-3) C > A, p.V637M				

M, Male; F, Female; AD, Atopic dermatitis; Eo, Eosinophilic esophagitis; GI, Gastrointestinal; OM, Otitis media; ABPA, Allergic bronchopulmonary aspergillosis; PJP, Pneumocystis Jirovecii Pneumonia; NA, Data is not available.

IgE levels of P1 decreased following dupilumab treatment. Reduced IgE levels were previously shown in dupilumab-treated patients, as demonstrated in a meta-analysis of seven studies (39). Published reports of DN-*STAT3* patients treated with dupilumab with subsequent IgE reduction further support that notion (15, 17).

Previous studies also support dupilumab treatment of atopic dermatitis in non-*STAT3*-related-IEI, such as DOCK8 deficiency (40), CARD11-associated atopy with dominant interference of NF-kB signaling (CADINS) (41), Wiskott-Aldrich Syndrome (42), common variable immune deficiency (CVID) (43), TTC7A-associated combined immunodeficiency (44), and X-linked agammaglobulinemia (45–47). Thus, indications for dupilumab use in IEI are rapidly expanding, and it should be considered a main therapeutic modality in adult patients with AD-HIES along with standard topical care.

Our study has several limitations, mainly its small number of patients and retrospective design. Nevertheless, we hope this cohort of adult AD-HIES patients will shed further light on the diagnosis and treatment of these patients.

In conclusion, immunologists and dermatologists treating AD-HIES patients should be aware of demodicosis as a possible clinical manifestation. Chronic and recalcitrant dermatophytosis may respond, at least partially, to voriconazole. Treatment with dupilumab appears to be safe and effective without need for systemic immunosuppressive agents and should be offered to these patients to alleviate their atopic dermatitis.

## Data availability statement

The original contributions presented in the study are included in the article/supplementary materials, further inquiries can be directed to the corresponding author.

## Ethics statement

The studies involving human participants were reviewed and approved by Hadassah Medical Center IRB. Written informed consent for participation was not required for this study in accordance with the national legislation and the institutional requirements.

## Author contributions

OS- Treatment of patients, study design and writing of the manuscript. LR- treatment of patients and manuscript revisions. AS- genetic and immune workup. AL – immune workup. OB- genetic workup. RS- immune workup. TF- immune workup, DH- immune workup and manuscript revisions. BG- manuscript revisions and treatment of patients. AN- immune workup,VP – dermatologic consultation, treatment of patients and study supervision, YT – study conceptualization and supervision. All authors contributed to the article and approved the submitted version.
